# Assessing the learning curve in laparoscopic TAPP repair for emergency inguinal hernia: a comparative study with open repair

**DOI:** 10.1038/s41598-026-64160-0

**Published:** 2026-08-05

**Authors:** Mohamed Wael, Islam El-Sayes

**Affiliations:** 1 Alexandria Main University Hospital, Alexandria, Egypt; 2 Faculty of Medicine, Alexandria University, Alexandria, Egypt

**Keywords:** Emergency inguinal hernia, Laparoscopic repair, TAPP, Learning curve, Open tension-free repair, Health care, Health occupations, Medical research, Signs and symptoms

## Abstract

Emergency inguinal hernia repair presents considerable surgical challenges, with a continuous debate regarding the best approach. This study analyzes the outcomes of laparoscopic transabdominal preperitoneal (TAPP) repair in comparison to open tension-free repair, while also assessing the learning curve associated with TAPP. A retrospective study was conducted on 71 patients undergoing emergency inguinal hernia repair (33 TAPP, 38 open). Outcomes included operative time, intraoperative complications, pain scores, hospital stay, return to activity, recurrence, and learning curve analysis comparing early (first 18) vs. late (last 15) TAPP cases. Operative time was longer with TAPP (98.3 ± 9.4vs.80.3 ± 11.4 min, *p* = 0.006), with a 6.1% conversion rate. Visceral injuries were more common in TAPP (15.2%vs.2.6%, *p* = 0.058), while vascular injuries were higher in open repair (10.5%vs.3.0%, *p* = 0.218). TAPP had significantly lower pain scores at 6, 12, and 24 h (*p* < 0.05), shorter hospital stay (2.5 ± 0.5vs.4.8 ± 0.8 days, *p* = 0.001), and earlier return to activities (5.5 ± 0.5vs.10.5 ± 1.0 days, *p* = 0.001). Recurrence and readmission rates were comparable. Later TAPP cases showed shorter operative time (93.4 vs. 102.4 min, *p* = 0.002), no conversions, and better pain control. TAPP is a feasible emergency repair option, for selected cases, with a clear learning curve which improves outcomes after 15–18 cases. However, larger prospective studies are required to confirm these findings

## Introduction

Inguinal hernias are among the most common surgical emergencies, often requiring urgent repair due to complications like incarceration, obstruction, or strangulation. While elective repairs are routine, the best surgical method for emergencies is still debatable^1^.

Historically, open tension-free repair was standardized. Nevertheless, developments in minimally invasive surgery have brought forth laparoscopic methods, especially transabdominal preperitoneal (TAPP) repair, as effective options even in emergency situations. There is a continuous discussion regarding the preferred method. Although many peer-reviewed studies indicate the benefits of lower postoperative complication rates, shorter hospital stays, reduced costs, decreased recurrence rates, less postoperative pain, quicker return to work, and enhancements in quality of life, and show that these techniques can be safely executed by surgeons globally, the uptake of laparoscopic techniques has been restricted until recently^2,3^

Laparoscopic repair offers advantages such as reduced postoperative pain, shorter hospital stays, fewer wound infections, and faster recovery^4^.

According to the 2017 World Society of Emergency Surgery (WSES) guidelines, laparoscopy can aid in assessing bowel viability post-reduction^5^. Several studies have demonstrated the feasibility of TAPP and totally extraperitoneal (TEP) repairs for incarcerated hernias under specific conditions. Nonetheless, concerns persist regarding their use in emergencies involving strangulation or bowel compromise, and current guidelines remain cautious^6^. Saye and Hollier^7^ were the first to report a modified preperitoneal femoral hernia repair with mesh fixation and resection of incarcerated small bowel under laparoscopic guidance in 1993. After that, several studies suggested that laparoscopic repair might be advantageous in specific emergency cases when selected with caution.

Several recent studies have evaluated the learning curve of TAPP hernia repair, primarily in elective settings. For instance, Özel et al.^8^ analyzed learning curves in TAPP herniorrhaphy and reported stabilization after 19–26 cases based on operative time and complication rates. However, their study focused exclusively on elective unilateral hernias and excluded emergency cases where technical difficulty, bowel viability assessment, and complication risk are significantly higher^9,10^.

This research aims to assess the safety, feasibility, and learning curve associated with TAPP repair in comparison to open tension-free repair for emergency inguinal hernia cases.

## Materials and methods

### Study design

This retrospective non-randomized comparative study was conducted at Alexandria Main University Hospital over a two-year period (2021–2022). It included adult patients who underwent emergency inguinal hernia repair using either laparoscopic TAPP or open tension-free techniques.

### Patients selection

Patients were categorized into two groups (TAPP versus open repair) based on the surgeon preference of the surgical approach performed for emergency inguinal hernia repair, patient condition and the availability of laparoscopic resources at the time of presentation.

Group I (TAPP, *n* = 33): Laparoscopic transabdominal preperitoneal repair.

Group II (Open, *n* = 38): Open tension-free repair via inguinal incision.

Patients were included if they met the following criteria: (a) Patients with confirmed diagnosis of an unilateral emergency inguinal hernia (incarcerated, obstructed, or strangulated) via clinical and radiological assessment (ultrasound or CT scan, if available). (b) Patients who underwent either TAPP repair or open tension-free repair for their emergency hernia condition. (c) Complete perioperative and follow-up data available.

Cases were divided chronologically into early and late phases to reflect progressive experience acquisition, a commonly used approach in surgical learning curve studies.

Patients with the following criteria were excluded; (a) Elective repairs performed during the study period. (b) Patients with missing, incomplete perioperative data as well as those with missing follow-up data were excluded (c) Emergency hernia cases presenting with strangulation and sure clinical/ radiological signs of perforation. (d) Patients with complicated recurrent inguinal hernia.

Patients initially planned for laparoscopic repair but converted to open intraoperatively were analyzed in the TAPP group (intention-to-treat analysis) to maintain study integrity.

### Ethical considerations

The study was approved by the Alexandria University Ethical Committee on Human Research (IRB Number: 00012098), and the requirement for informed consent was waived due to the retrospective nature of the study. Data protection and patients’ privacy were guaranteed by the authors.

### Study outcomes

The aim of this retrospective study is to assess the safety and feasibility of the TAPP technique for managing inguinal hernias in emergency situations, comparing this minimally invasive approach with the open surgical method. A learning curve analysis was performed for the TAPP technique by comparing early cases (first 18 cases) with late cases (last 15 cases), to evaluate changes in operative performance over time. A similar temporal comparison was conducted in the open group for reference.

### Preoperative management

All patients underwent assessment in the Emergency Department by the on-call resident to diagnose the emergency indication. The decision for surgical intervention was made at the surgeon’s discretion, favoring the TAPP technique unless contraindicated/ not available. An indication algorithm for surgical technique choice was not utilized, as one has not yet been established for emergency hernia repairs^11^.

Prior to proceeding, informed consent for hernia surgical repair including a detailed explanation of all techniques employed.

### Surgical management

All patients were administered perioperative prophylactic antibiotics together with antiemetic at induction as well as general anesthesia with endotracheal intubation. All procedures were conducted in our surgical tertiary center by the same experienced surgical team (two senior consultants and a senior resident) skilled in both laparoscopic and open surgical techniques.

#### Group I: *TAPP repair (laparoscopic approach)*

The majority of the patients received TAPP repair, as this is the primary laparoscopic repair employed and taught by our department. Generally, the factors influencing for laparoscopic repair were the availability of laparoscopic settings upon patients’ presentation, the patient’s hemodynamic condition and fitness for pneumoperitoneum, and the patient’s lack of a previous lower abdominal surgery.

In the trendelenberg position, pneumoperitoneum was using the Hasson technique^12^. After careful diagnostic laparoscopy, two more 5-mm trocars were placed in the right and left flank. The peritoneum was incised transversely above the inguinal ring, the hernia sac was reduced by dissecting the hernia sac from any adhesions avoiding excessive traction, and a 10 × 15 cm ULTRAPRO mesh (Ethicon Endo-Surgery) mesh was the most commonly used being placed in the preperitoneal space. The parietal peritoneum was sutured with running self-locking sutures.

Laparoscopic assessment of bowel status was conducted. If bowel resection becomes necessary, bowel manipulation occurs only after the prosthesis is positioned and the parietal peritoneum is closed. If bowel resection was required, it was performed with laparoscopic instruments/staplers and the specimen was then brought out through an endoscopic retrieval bag to prevent dissemination of infection. If not feasible, resection and anastomosis of the bowel were then performed extracorporeally. Hamostasis was ensured and trocar sites were closed appropriately^13^.

#### Group II: open tension-free repair

Open repair used a standard Lichtenstein tension-free approach through a 5–8 cm inguinal incision, with mesh placement after hernia sac management. Bowel resection, if needed, was performed through the same incision.^14,15^.

### Follow-up

Follow-up took place in the outpatient clinic at scheduled visits (reaching 18 months) Any instances of complications or early recurrence were documented. The patients who did not attend their scheduled clinic appointments were considered lost to follow-up and were excluded from the analysis.

### Statistical analysis

Statistical analysis was conducted utilizing IBM SPSS. Categorical data were analyzed using chi-square or Fisher’s exact test; continuous variables with t-test or Mann–Whitney U test. *p* ≤ 0.05 was considered significant. Data are presented as means ± standard deviation for normally distributed variables, and as median and interquartile range for those that are not normally distributed.

No prior sample size calculation was performed, as this was a retrospective study including all eligible emergency cases presenting during the study period. Given the relatively low incidence of emergency inguinal hernias managed laparoscopically, all consecutive patients over the two-year period were included.

A post hoc power analysis was performed based on the observed difference in operative time between groups using a two-tailed independent-samples t-test with *α* = 0.05. The achieved power was high, suggesting that the available sample size was sufficient to detect differences in the primary outcome (operative time) 

## Results

A total of 71 patients underwent emergency inguinal hernia repair: 33 underwent TAPP and 38 underwent open tension-free repair.

- Baseline characteristics were comparable between groups.

1. Demographics, ASA Classification and Comorbidity Burden of the included patients (Table 1): Mean age was similar (TAPP: 41.3 ± 13.6 vs. Open: 44.7 ± 13.6 years, *p* = 0.149). Most patients were male (TAPP: 69.7%, Open: 57.9%, *p* = 0.303). BMI was higher in the TAPP group (35.9 ± 3.7 vs. 34.1 ± 3.3, *p* = 0.019) which may suggest a preference for laparoscopic repair in obese patients due to concerns over wound healing in open surgery. ASA classifications and Charlson Comorbidity Index (CCI) scores showed no significant differences (*p* = 0.246 and 0.065, respectively).
2. Clinical presentations (Table 2): Both groups had comparable clinical severity at presentation; Obstruction occurred in 39.4% (TAPP) vs. 57.9% (Open), *p* = 0.119. Strangulation was present in 24.2% (TAPP) vs. 31.6% (Open, *p* = 0.493).).

**Table 1:** Comparison between the two studied groups regarding basic demographic

	“TAPP” “n=33”		“Open” “n=38”		Test *P* value
	No	%	No	%	
Age Range Mean SD	20–70 41.3 13.6		21–65 44.7 13.6		T=1.26 0.149 N.S.
Sex Male Female	23 10	69.7 30.3	22 16	57.9 42.1	X^2^ 1.06 0.303 N.S.
BMI Range Mean SD	30.2–42.2 35.9 3.7		28.1–38.8 34.1 3.3		T=2.09 0.019*
ASA score I II III	21 7 5	63.6 21.2 15.2	18 15 5	47.4 39.5 13.2	X^2^ 2.801 0.246 N.S.
CCI Range Mean SD	1.3–3.3 2.3 0.6		1.6–3.6 2.6 0.7		0.065

T= student t-test

X^2^ = Chi square test

P was significant if ≤ 0.05

N.S. = Not significant

^*^Significant difference at 0.05

**Table 2:** Comparison between the two studied groups regarding clinical presentation

	“TAPP” “n=33”		“Open” “n=38”		Test *P* value
	No	%	No	%	
Onset (hours) Range Mean SD	9–68 35.0 18.4		6–70 32.9 19.7		T=0.985 0.324
Cases with Sure signs of obstruction No Yes	20 13	60.6 39.4	16 22	42.1 57.9	X^2^ 2.418 0.119
Cases with acute incarceration No Yes	25 8	75.8 24.2	26 12	68.4 31.6	X^2^0.469 0.493

T= student t-test

X^2^ = Chi square test

P was significant if ≤ 0.05

N.S. = Not significant

(b) Intraoperative parameters (Table 3).

**Table 3:** Comparison between the two studied groups regarding operative data

Operative parameters	“TAPP” “n=33”		“Open” “n=38”		T test *P* value
	No	%	No	%	
Time (minutes) Range Mean SD	82–119 98.3 9.4		61–98 80.3 11.4		3.52 0.006*
Type of hernia Direct Indirect Combined	12 6 15	36.4 18.2 45.5	13 16 9	34.2 42.1 23.7	5.761 0.560
Number of defects 1 2	15 18	45.5 54.5	19 19	50.0 50.0	0.146 0.702
Size of defects Small <2cm Medium 2–4cm Large > 4cm	17 9 7	51.5 27.3 21.2	17 10 11	44.7 26.3 28.9	0.592 0.743
Cases with conversion to open surgery No Yes	31 2	93.9 6.1	38 0	100.0 0.0	0.547 0.459
Cases with Visceral injury No Yes	28 5	84.8 15.2	37 1	97.4 2.6	3.578 0.058
Cases with Vascular injury No Yes	32 1	97.0 3.0	34 4	89.5 10.5	1.516 0.218
Cases with Intestinal Resection done No Yes	31 2	93.9 6.1	35 3	92.1 7.9	0.090 0.763

*P* was significant if ≤ 0.05

1. Operative time: TAPP procedures took significantly longer time than open repairs (98.3 ± 9.4 vs. 80.3 ± 11.4 min, *p* = 0.006).
2. Conversion to Open Repair: Two TAPP cases (6.1%) required conversion—one due to difficulty reducing the hernia, another due to uncertain bowel viability (*p* = 0.459).
3. Visceral Injury: TAPP group had a higher rate (15.2% vs. 2.6%, *p* = 0.058). Injuries included serosal tears (bowel and colon), bladder injury, enterotomy, and trocar-related bowel trauma. Open group had one case of bowel contusion during manual reduction, which was managed conservatively without ischemic changes necessitated resection.
4. Vascular Injury: Observed more in the open group (10.5% vs. 3.0%, *p* = 0.218). Open injuries involved the inferior epigastric artery and pampiniform plexus; TAPP injuries were trocar-related.
5. Assessment of bowel viability and intestinal resection: Acutely incarcerated hernias were comparable between the TAPP and open groups (24.2% vs. 31.6%, *p* = 0.493). Despite this, intestinal resection was required in 2 patients in the TAPP group and 3 patients in the open group (*p* = 0.763), all of them were ischemic non perforated.

(c) *Postoperative pain assessment* Postoperative pain assessment at 6, 12, and 24 h (Table 4) showed significantly lower pain scores in the TAPP group across all time points (*p* < 0.005). This may support the advantages of laparoscopic surgery in reducing early postoperative discomfort.

**Table 4:** Comparison between the two studied groups regarding pain score

Pain score	“TAPP” “n=33”	“Open” “n=38”	T test P value
6h Range Mean SD	3–5 4.0 0.8	4–8 6.2 1.5	3.98 0.002*
12h Range Mean SD	2 4 2.8 0.8	3 7 5.0 1.6	3.52 0.005*
24h Range Mean SD	1 3 1.9 0.8	2 4 3.4 0.8	3.44 0.003*

*P* was significant if ≤ 0.05

(d) * Postoperative Complications* (Table 5) Wound infection was more frequent, although non significant, in the open group (21.1% vs 6.1%, *p* = 0.07), suggesting a possible trend of laparoscopy to reduce wound-related complications by avoiding large incisions. Other complications, including scrotal edema, ileus, and chronic groin pain, showed no statistically significant differences between the groups (*p* > 0.1). No cases of postoperative mortality were documented in both groups.

**Table 5:** Comparison between the two studied groups regarding incidence of post-operative complications

Complications	“TAPP” “n=33”		“Open” “n=38”		X^2^ *P* value
	No	%	No	%	
Wound infection No Yes	31 2	93.9 6.1	30 8	78.9 21.1	3.280 0.070
Scrotal edema No Yes	32 1	97.0 3.0	35 3	92.1 7.9	0.786 0.375
Urine retention No Yes	33 0	100.0 0.0	33 0	100.0 0.0	–
Ileus No Yes	30 3	90.9 9.1	36 2	94.7 5.3	0.395 0.529
Mesh infection No Yes	33 0	100.0 0.0	37 1	97.4 2.6	0.006 0.936
Death No Yes	33 0	100.0 0.0	38 0	100.0 0.0	–

*P *was significant if ≤ 0.05

(e) Postoperative recovery Outcomes (Table 6) Hospital stay and return to normal activities (Table 6) were significantly shorter in the TAPP group (p = 0.001), suggesting its role in faster recovery and earlier mobilization. Recurrence rates were comparable (3.0% in TAPP vs. 7.9% in Open, *p* = 0.375), suggesting that both techniques provide effective long lasting hernia repair (Table 7). Similarly, 30 day readmission and reoperation rates were low with no significant differences (*p* > 0.9) between both groups (Table 8). These findings may reflect that TAPP is an effective alternative to open repair, maintaining comparable long-term outcomes without increasing the risk of recurrence or reoperation.

(f) Learning Curve Analysis in TAPP vs. Open Repair To evaluate the learning curve for TAPP repair, outcomes from the first 18 cases (early group) were compared to the last 15 cases (late group). Significant improvements were observed across key intraoperative and postoperative parameters as surgical experience increased.

**Table 6:** Comparison between the two studied groups regarding hospital stay, recurrence, readmission and reoperation rates

	“TAPP” “n=33”		“Open” “n=38”		X^2^ P value
	No	%	No	%	
Recurrence No Yes	32 1	97.0 3.0	35 3	92.1 7.9	0.786 0.375
30 day readmission No Yes	33 0	100.0 0.0	37 1	97.4 2.6	0.006 0.936
Reoperation No Yes	33 0	100.0 0.0	37 1	97.4 2.6	0.006 0.936
Hospital stay (days) Range Mean SD	2–3 2.5 0.5		4–6 4.8 0.8		0.001*
Return to normal activities (days) Range Mean SD	5–6 5.5 0.5		9-12 10.5 1.0		0.001*

*P *was significant if ≤ 0.05

**Table 7:** Learning curve analysis across different parameters

Operative	“TAPP” “n=33”				“Open” “n=38”			
	Early cases “n=18”		Late cases “n=15”		Early cases “n=19”		Late cases “n=19”	
*Time*								
Range	89.0–119.0		82–110		61–98		63–98	
Mean	102.4		93.4		79.6		81.0	
SD	8.7		7.9		13.5		9.2	
*P* value	0.002*				0.265			
*Conversion to open*								
No	16	88.9	15	100.0	19	100.0	19	100.0
Yes	2	11.1	0	0.0	0	0.0	0	0.0
*P* value	0.039*				–			
*Visceral injury *								
No	15	83.3	13	86.7	18	94.7	19	100.0
Yes	3	16.7	2	13.3	1	5.3	0	0.0
*P* value	0.399							
*Vascular injury *								
No	18	100.0	14	93.3	17	89.5	17	89.5
Yes	0	0.0	1	6.7	2	10.5	2	10.5
*P* value	0.56				1.00			
*Intestinal Resection *								
No	17	94.4	14	93.3	17	89.5	18	94.7
Yes	1	5.6	1	6.7	2	10.5	1	5.2
*P* value	0.449				0.089			

*P *was significant if ≤ 0.05

**Table 8:** Comparison between the early and late groups regarding pain score

Pain score	“TAPP” “n=33”		“Open” “n=38”	
	Early “n=18”	Late “n=15”	Early “n=19”	Late “n=19”
6h				
Range	3.0–5.0	3–5	4–8	4–8
Mean	4.2	3.7	5.7	6.6
SD	0.7	0.8	1.3	1.5
*P* value	0.042*		0.068	
12h				
Range	2.0–4.0	2–3	3–7	3–7
Mean	3.2	2.3	5.3	4.7
SD	0.9	0.5	1.7	1.6
*P* value	0.022*		0.103	
24h				
Range	1.0–3.0	1–2	2–4	2–4
Mean	2.4	1.3	3.4	3.4
SD	0.7	0.5	0.77	0.81
*P* value	0.031*		0.98	

*P *was significant if ≤ 0.05

Intraoperative learning curve trends (Table 7)

**Fig. 1:**
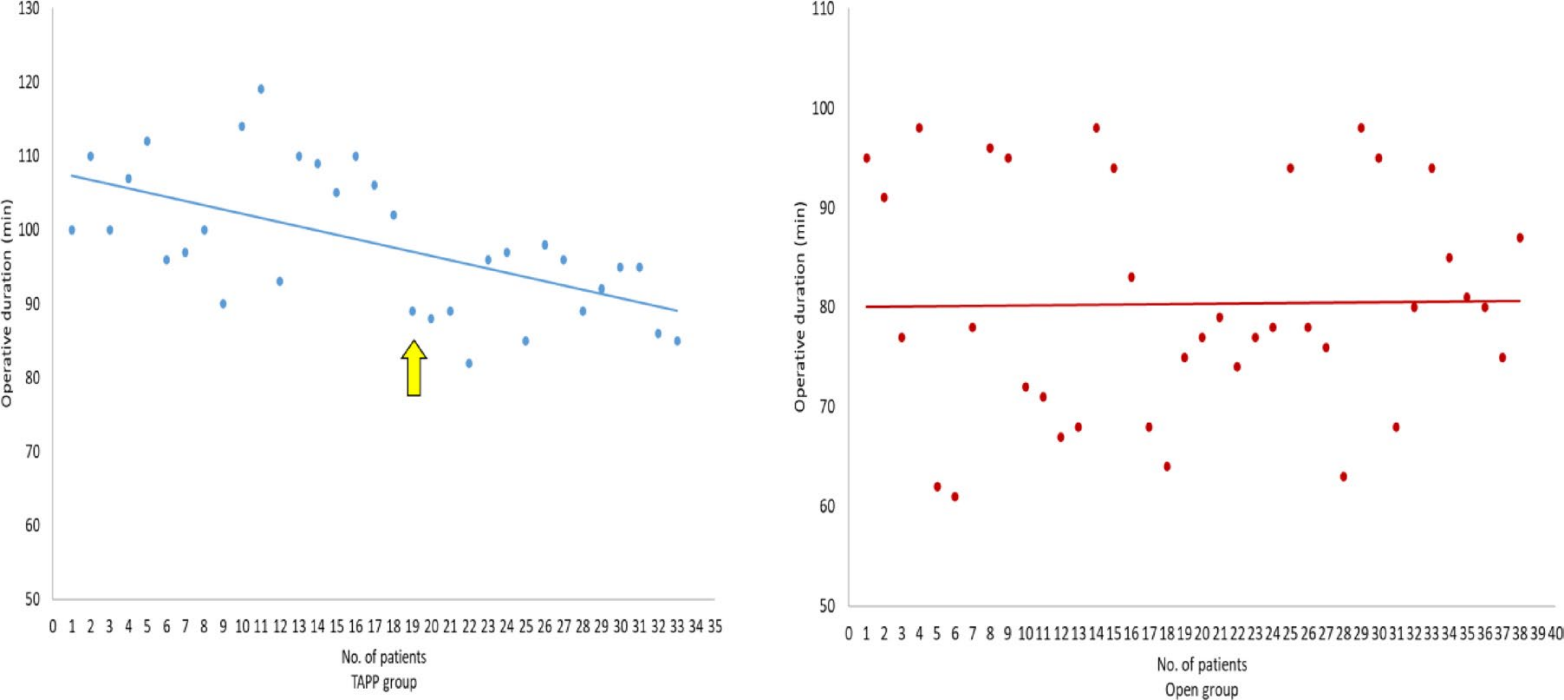
CUSUM analysis showing trend of operative time across consecutive TAPP cases. A noticeable change in slope is observed around case 18, suggesting a transition from the initial learning phase to a more stable performance phase. b: CUSUM analysis showing trend of operative time across consecutive open cases.

1. Operative time in the TAPP group decreased notably with experience, from 102.4 ± 8.7 min in early cases to 93.4 ± 7.9 min in late cases (*p* = 0.002). A graphical trend analysis of operative time across consecutive TAPP cases (Fig. 1) demonstrated a gradual reduction over time, with a noticeable change in slope around case 18, reflecting improved efficiency in dissection, mesh handling, and closure techniques. In contrast, operative time remained stable in the open group (79.6 ± 13.5 vs. 81.0 ± 9.2 min, *p* = 0.265).
2. Conversion to Open Surgery: In early TAPP cases, 11.1% required conversion to open repair, primarily due to difficulty in reducing incarcerated hernias or concerns over bowel viability. However, in late cases, the conversion rate dropped to 0% (*p* = 0.039).
3. Visceral and Vascular Injuries: Visceral injuries in TAPP slightly declined with experience (16.7% early vs. 13.3% late, *p* = 0.399, NS). Though not statistically significant, this suggests safer dissection techniques over time. In the open group, visceral injury rates were low and unchanged (5.3% early vs. 0% late, *p* = 0.84). Vascular injuries in TAPP were rare, with only one case in the late group (6.7%), and none in the early group. Open repair showed comparable non significant variation (10.5% early vs. 10.5% late, *p* = 1.00).

Postoperative learning curve trends (Tables 8 and 9)

**Table 9:** Comparison between the early and late groups regarding hospital stay and duration of return to normal activities

	“TAPP” “*n* = 33”		“Open” “*n* = 38”	
	Early “*n* = 18”	Late “*n* = 15”	Early “*n* = 19”	Late “*n* = 19”
Hospital stay (days) Range Mean SD	2.0–3.0 2.8 0.4	2–3 2.1 0.4	4–6 4.7 0.7	4–6 4.9 0.8
*P* value	0.040*		0.65	
Return to normal activities (days) Range Mean SD	5.0–6.0 5.7 0.5	5–6 5.3 0.5	9–12 10.2 0.9	9–12 10.7 1.0
*P* value	0.103		0.55

1. Postoperative pain scores in the TAPP group improved significantly. At 6 h, VAS scores dropped from 4.2 ± 0.7 in early cases to 3.7 ± 0.8 in late cases (*p* = 0.042). At 12 h, scores decreased from 3.2 ± 0.9 to 2.3 ± 0.5 (p = 0.022), and at 24 h, from 2.4 ± 0.7 to 1.3 ± 0.5 (*p* = 0.031). These improvements likely reflect gentler tissue handling and better procedural flow. In contrast, the open group showed no significant change in VAS scores at any time point (all *p* > 0.05).
2. Hospital stay also decreased with TAPP experience, from 2.8 ± 0.4 days in early cases to 2.1 ± 0.4 days in late cases (*p* = 0.040). This was likely due to reduced operative time, fewer complications, and improved postoperative management. In the open group, hospital stay remained stable (4.7 vs. 4.9 days, *p* = 0.65).
3. Return to normal activities showed a favorable trend in late TAPP cases (5.3 ± 0.5 days) compared to early cases (5.7 ± 0.5 days), although this was not statistically significant (*p* = 0.103). The open group showed no improvement over time (10.2 ± 0.9 vs. 10.7 ± 1.0 days, *p* = 0.55).

## Discussion

In recent decades, there has been a consistent shift in surgery towards minimally invasive techniques, which provide advantages like decreased pain, faster recovery times, and shorter hospital admissions. Specifically, in the realm of inguinal hernia repair, laparoscopy has become more widely accepted, especially for bilateral and recurrent hernias, as this minimally invasive method offers improved anatomical visualization and reduced postoperative discomfort^16,17^. However, the role of laparoscopic repair in emergency inguinal hernias remains debated due to concerns over technical difficulty, longer operative time, and limited exposure in cases of strangulation or bowel compromise ^18–20^.

Latest WSES guidelines^5^ are still cautious about this topic recommending laparoscopy for assessment of bowel viability and the HerniaSurge group^11^ underline that no randomized study focus on laparoscopic technique in emergency.

This study contributes to the growing body of evidence evaluating the feasibility, safety, and effectiveness of laparoscopic TAPP repair in emergency settings as well as the learning curve, comparing its outcomes with traditional open tension-free repair. This study is a retrospective comparative analysis, meaning that patients were not randomly assigned to either TAPP or open repair, but rather underwent surgery based on surgeon preference, clinical presentation, and patient factors as well as the availability of laparoscopic settings. While randomized controlled trials are the gold standard for comparing surgical techniques, retrospective studies provide valuable real-world insights into the feasibility and safety of different approaches in emergency settings, where strict randomization is often impractical.

The baseline characteristics of the two groups were comparable, ensuring that differences in outcomes were more likely related to the surgical approach rather than patient selection bias. However, the higher BMI in the TAPP group (*p* = 0.019) suggests a possible preference for laparoscopic repair in obese patients, potentially due to concerns over wound healing and infection risks in open surgery. Additionally, ASA score was comparable (*p* = 0.246), confirming that both groups had a similar perioperative risk profile. These findings suggest that neither technique was disproportionately applied to higher-risk patients, strengthening the validity of the outcome comparisons.

The longer operative time observed in the TAPP group aligns with existing literature^21^, where laparoscopic procedures often require more meticulous dissection and additional steps such as peritoneal closure and mesh placement. However, with surgeon experience and optimized techniques, operative times tend to improve, as seen in elective laparoscopic hernia repairs.

Acutely incarcerated hernias were comparable between the TAPP and open groups (24.2% vs. 31.6%, *p* = 0.493). Despite this, intestinal resection was required in only a small number of cases: 2 (6.1%) in the TAPP group and 3 (7.9%) in the open group (*p* = 0.763), all of them were ischemic non perforated. Cases with preoperative clinical/radiological signs of bowel perforation were excluded from the study and were operated by the open approach from the start. All resections were performed safely without need for additional incisions and mesh was inserted before bowel resection with peritoneal mesh coverage before ischemic bowel manupulation to prevent mesh contamination. The 6.1% conversion rate to open surgery in the TAPP group is consistent with reported rates in emergency settings^10^, highlighting the technical challenges of minimally invasive repair in complicated hernias.

In our study, the relatively high visceral injury rate in the TAPP group (15.2%) is a notable finding and appears higher than rates reported in the literature (1–3%)^11,20^. This may be explained by the early learning phase, the emergency nature of the selected cases, which often involve bowel distension, adhesions, and compromised tissues, increasing the technical difficulty of laparoscopic dissection. Importantly, most injuries were minor (e.g., serosal tears and trocar-related trauma) and did not result in significant clinical consequences.

Therefore, this result should be interpreted cautiously and highlights the importance of surgical expertise and appropriate case selection. In contrast, more vascular injury was reported in the open group (10.5% vs. 3.0%, *p* = 0.218), likely due to direct dissection of the hernia sac, particularly near the inferior epigastric vessels and spermatic cord. These findings underscore the importance of surgical expertise in minimizing intraoperative complications, particularly when selecting TAPP for complex emergency cases.

Chronic groin pain is a clinically relevant long-term outcome after inguinal hernia repair. Possible contributing factors include mesh fixation methods and nerve irritation within the preperitoneal space, particularly with tacker use or dissection near neural structures in our study, it was higher in the TAPP group (12.1% vs. 2.6%), although the difference was not statistically significant. This aligns with the established benefits of laparoscopic surgery, where smaller incisions and reduced tissue trauma contribute to improved pain control.

Hospital stay was significantly shorter in the TAPP group (2.5 ± 0.5 days) compared to the open group (4.8 ± 0.8 days, *p* = 0.001) Likewise, individuals in the TAPP group resumed their normal activities significantly sooner (5.5 ± 0.5 vs. 10.5 ± 1.0 days, *p* = 0.001). These results are consistent with existing evidence that endorses laparoscopy as a method to improve postoperative recovery^22–24^. In emergency settings, less postoperative pain earlier mobilization and reduced hospitalization may be particularly beneficial in reducing hospital-associated complications.

The wound infection rate, although being not statistically significant, was slightly more in the open group (21.1%) compared to the TAPP group (6.1%, *p* = 0.07, borderline significance). This may indicate that laparoscopic surgery minimizes wound exposure and reduces the risk of infection, to be further evaluated by more studies with more sample size. Other complications, such as scrotal edema (3.0% vs. 7.9%, *p* = 0.375) and postoperative ileus (9.1% vs. 5.3%, *p* = 0.529), showed no significant differences, indicating comparable safety profiles. Chronic groin pain was slightly higher in the TAPP group (12.1%) compared to open repair (2.6%, *p* = 0.119), which may be related to mesh fixation techniques and nerve irritation in the preperitoneal space.

Both methods showed low comparable rates of recurrence (3.0% for TAPP compared to 7.9% for Open, *p* = 0.375), indicating that when conducted by skilled surgeons, TAPP repair is as effective as open repair in preventing recurrence. Additionally, 30-day readmission (0% vs. 2.6%, *p* = 0.936) and reoperation rates (0% vs. 2.6%, *p* = 0.936) were low in both groups. These findings are consistent with prior studies that report equivalent recurrence rates between laparoscopic and open hernia repair when proper technique and mesh placement are ensured^10,21,22^.

The cases analyzed in our research were categorized chronologically into early and late phases to illustrate the gradual acquisition of experience, a widely accepted approach for assessing surgical learning curves. In our study, the observed transition around case 18 may represent a threshold at which basic technical proficiency is achieved. The noted enhancement in operational efficiency and postoperative results indicates a tangible learning curve effect in laparoscopic TAPP repair for emergency inguinal hernias. A systematic review and meta-analysis by Sivakumar, Chen^25^ estimated that surgical proficiency in laparoscopic inguinal hernia repair is typically achieved after 32.5 cases. Similarly, a study by Gao, Zeng^26^ using cumulative sum (CUSUM) analysis found that proficiency in elective TAPP repair was achieved after 30 cases, further supporting our findings that learning progression is measurable and leads to better surgical outcomes.

The earlier proficiency observed in our study compared with elective series may reflect prior surgeon experience with laparoscopic techniques, the focused exposure during emergency cases, and standardized operative workflow rather than a true reduction in the learning requirement.

Conversely, in our open repair group, there was no significant difference in operative time, complications, or length of hospital stay between early and late cases, suggesting that open repair does not exhibit a learning curve effect. This is consistent with the general understanding that open hernia repair is a more standardized procedure, with outcomes that remain stable regardless of case sequence.

The operative time for TAPP repair significantly decreased from 102.4 ± 8.7 min in early cases to 93.4 ± 7.9 min in late cases (*p* = 0.002), indicating that surgical proficiency improved as case volume increased. This trend is well-documented in previous studies on elective laparoscopic hernia repair^26^. Interestingly, our findings suggest that proficiency in emergency TAPP repair may be achieved earlier, around 15–18 cases, possibly due to the intensified learning experience in urgent cases. In contrast, open repair operative time remained stable (81.1 ± 10.9 min for late cases vs. 79.5 ± 11.8 min for early cases, *p* = 0.62). This may reflect that open surgery does not require the same level of skill refinement over time.

Conversion to open surgery was another key marker of skill progression in TAPP. The conversion rate significantly declined from 11.1% in early cases to 0% in late cases (*p* = 0.039), demonstrating that laparoscopic case selection and technical handling improve with experience. Since open repair does not require conversion, this further reinforces that TAPP outcomes are experience-dependent, whereas open surgery maintains a stable trend.

Postoperative outcomes also demonstrated a clear learning effect in TAPP repair, with improvements in pain control, hospital stay, and early recovery in late cases.

The duration of hospital stays for TAPP repair has significantly reduced from 2.8 ± 0.4 days in earlier cases to 2.1 ± 0.4 days in later cases (*p* = 0.040, significant). This indicates that subsequent cases experienced advantages such as shorter operative times, fewer complications, and improved perioperative management. In contrast, the length of hospital stay for the open repair group remained consistent (4.9 vs. 4.7 days, *p* = 0.58), highlighting that open surgery does not demonstrate a learning curve in terms of postoperative recovery.

Pain control also improved with surgeon experience in TAPP repair, as VAS scores at 6 h decreased significantly from 4.2 ± 0.7 to 3.7 ± 0.8 (*p* = 0.042), and at 12 h from 3.2 ± 0.9 to 2.3 ± 0.5 (*p* = 0.022). However, in the open repair group, postoperative pain remained stable between early and late cases (VAS 6 h: 6.4 ± 1.6 vs. 6.1 ± 1.3, *p* = 0.47) with no significant difference. This may support that open surgery does not follow a learning curve trajectory. Although the differences in postoperative pain scores were statistically significant, their clinical relevance was further evaluated using the minimum clinically important difference (MCID) for VAS, typically reported as 1.3–2.0 points. In our study, the observed difference (e.g., 2.2 points at 6 h) exceeds this threshold, suggesting that the reduction in pain is not only statistically significant but also clinically meaningful. These findings suggest that as laparoscopic surgeons become more proficient, they develop techniques that reduce tissue handling and minimize postoperative pain.

While return to normal activities was slightly faster in late TAPP cases (5.3 ± 0.5 vs. 5.7 ± 0.5 days, *p* = 0.103), this trend was not statistically significant. In contrast, open repair recovery remained unchanged (10.6 vs. 10.4 days, *p* = 0.76), suggesting that TAPP benefits from progressive learning, while open surgery outcomes remain constant regardless of surgeon experience.

This study has several strengths. First, it is one of the few studies to evaluate laparoscopic TAPP repair specifically in emergency inguinal hernia. Second, our study provides a direct unique comparison between early and late TAPP cases with subsequent interpretation of the surgeon learning curve in managing such emergency cases, demonstrating a significant reduction in operative time (*p* = 0.002), conversion rates (*p* = 0.039), and hospital stay (*p* = 0.040), reinforcing that experience plays a crucial role in optimizing laparoscopic outcomes.

This study has several limitations; the retrospective design introduces inherent selection bias, as the choice of surgical approach was influenced by surgeon preference, patient condition, and the availability of laparoscopic resources. Another limitation is the significantly higher BMI noted in the TAPP group, which may indicate a selection bias favoring laparoscopic repair in obese patients due to concerns about wound complications associated with open surgery. This difference could serve as a confounding factor that impacts both perioperative outcomes and postoperative recovery.

Although the emergency nature of the study with the rarity of included cases demonstrated a significant difference in operative time with high achieved power, it remains underpowered for detecting differences in low-frequency. Accordingly, due to the limited sample size, the study remains underpowered for outcomes such as visceral injury, recurrence and mesh-related complications. Although no patients were lost in the follow up, the follow-up period of 18 months may not be adequate to thoroughly assess long-term recurrence rates.

This study is initially exploratory in the concern of emergency inguinal hernia repair and future multicenter prospective studies should focus on longer-term outcomes and standardizing training protocols to determine how surgeon proficiency can be accelerated in emergency laparoscopic hernia repair.

## Conclusion

Laparoscopic TAPP repair appears to be a feasible option for selected emergency inguinal hernia cases and demonstrates trends toward improved recovery outcomes. In our study, a learning curve effect was observed, with improved performance after approximately 18 cases. However, prospective studies with larger sample size are required to confirm these findings**.**

## Data Availability

The data that support the findings of this study are available from Alexandria Main University Hospital but restrictions apply to the availability of these data, which were used under institutional license for the current study and are therefore not publicly available. However, the data are available from the corresponding author on reasonable request and with permission from Alexandria Main University Hospital.
